# Chytrid infections exhibit historical spread and contemporary seasonality in a declining stream-breeding frog

**DOI:** 10.1098/rsos.231270

**Published:** 2024-01-31

**Authors:** A. M. Belasen, R. A. Peek, A. J. Adams, I. D. Russell, M. E. De León, M. J. Adams, J. Bettaso, K. G. H. Breedveld, A. Catenazzi, C. P. Dillingham, D. A. Grear, B. J. Halstead, P. G. Johnson, P. M. Kleeman, M. S. Koo, C. W. Koppl, J. D. Lauder, G. Padgett-Flohr, J. Piovia-Scott, K. L. Pope, V. Vredenburg, M. Westphal, K. Wiseman, S. J. Kupferberg

**Affiliations:** ^1^ Department of Integrative Biology, University of Texas at Austin, Austin, TX, USA; ^2^ Department of Ecology and Evolutionary Biology, Cornell University, Ithaca, NY, USA; ^3^ California Department of Fish and Wildlife, West Sacramento, CA, USA; ^4^ Earth Research Institute, University of California, Santa Barbara, CA, USA; ^5^ Department of Ecology, Evolution, and Marine Biology, University of California, Santa Barbara, CA, USA; ^6^ Genome Center, University of California, Davis, CA, USA; ^7^ U.S. Geological Survey, Forest and Rangeland Ecosystem Science Center, Corvallis, OR, USA; ^8^ Six Rivers National Forest, Lower Trinity Ranger District, USDA Forest Service, P.O. Box 68, Willow Creek, CA, USA; ^9^ Spring Rivers Ecological Sciences LLC, Cassel, CA, USA; ^10^ Department of Biological Sciences, Florida International University, Miami, FL, USA; ^11^ Plumas National Forest, USDA Forest Service, Quincy, CA, USA; ^12^ U.S. Geological Survey, National Wildlife Health Center, Madison, WI, USA; ^13^ Point Reyes Field Station, U.S. Geological Survey, Western Ecological Research Center, Point Reyes Station, CA, USA; ^14^ Pinnacles National Park, National Park Service, Paicines, CA, USA; ^15^ Museum of Vertebrate Zoology, University of California, Berkeley, CA; ^16^ Department of Integrative Biology, University of California, Berkeley, CA, USA; ^17^ Sierra Streams Institute, Nevada City, CA, USA; ^18^ ICF, Sacramento, CA; ^19^ School of Biological Sciences, Washington State University, Vancouver, WA, USA; ^20^ Pacific Southwest Research Station, USDA Forest Service, Arcata, CA, USA; ^21^ Department of Biology, San Francisco State University, San Francisco, CA, USA; ^22^ Central Coast Field Office, United States Bureau of Land Management, Marina, CA, USA; ^23^ Department of Herpetology, California Academy of Sciences, San Francisco, CA, USA

**Keywords:** spatio-temporal disease model, amphibian chytridiomycosis, Mediterranean climate, seasonality, freshwater conservation, *Rana boylii*

## Abstract

Species with extensive geographical ranges pose special challenges to assessing drivers of wildlife disease, necessitating collaborative and large-scale analyses. The imperilled foothill yellow-legged frog (*Rana boylii*) inhabits a wide geographical range and variable conditions in rivers of California and Oregon (USA), and is considered threatened by the pathogen *Batrachochytrium dendrobatidis* (Bd). To assess drivers of Bd infections over time and space, we compiled over 2000 datapoints from *R. boylii* museum specimens (collected 1897–2005) and field samples (2005–2021) spanning 9° of latitude. We observed a south-to-north spread of Bd detections beginning in the 1940s and increase in prevalence from the 1940s to 1970s, coinciding with extirpation from southern latitudes. We detected eight high-prevalence geographical clusters through time that span the species' geographical range. Field-sampled male *R. boylii* exhibited the highest prevalence, and juveniles sampled in autumn exhibited the highest loads. Bd infection risk was highest in lower elevation rain-dominated watersheds, and with cool temperatures and low stream-flow conditions at the end of the dry season. Through a holistic assessment of relationships between infection risk, geographical context and time, we identify the locations and time periods where Bd mitigation and monitoring will be critical for conservation of this imperilled species.

## 1. Introduction

Threatened species with large geographical ranges often require unique, regional conservation strategies to combat stressors such as infectious disease. Pathogen surveys and reporting have become standard for North American wildlife diseases [1,2]; however, relative risk across a landscape and among populations within species remains difficult to anticipate, especially when data are collected by separate research groups [3]. Central reporting databases [4], synthetic analyses and retrospective surveys can help assess disease threats and identify high-risk populations.

Among the most significant wildlife diseases, amphibian chytridiomycosis caused by the fungal pathogen *Batrachochytrium dendrobatidis* (Bd) has contributed to declines of hundreds of species worldwide [5]; but see [6]. In North America, notable Bd-associated declines have occurred across the west including the southern Rocky Mountains [7,8], Arizona and New Mexico [9,10], Nevada [11] and California [12–14]. In several of these cases, infection outcomes varied widely among populations due to host-related and environmental factors including genetics, prior Bd exposure and abiotic conditions [15–17].

For the stream-dwelling foothill yellow-legged frog, *Rana boylii*, Bd's role in the species' changing abundance across its endemic range (California and Oregon, USA) is not well-understood. The species has declined for at least the last half-century, with extirpations reported from xeric lower latitudes [18], at the wetter northern range limit [19] and downstream of large dams range-wide [20]. A mix of abiotic and biotic factors influence Bd infection risk and disease dynamics in many systems, including elevation, latitude, climate, habitat quality and host characteristics [21]. The relative importance of these factors remains unclear in rivers with winter flood/summer drought flow regimes typical across *R. boylii*'s geographical range. Bd is considered a significant potential threat to *R. boylii* [22] because it is implicated in the species' disappearance from rivers of California's South Coast [23] and in recent autumn die-offs of *R. boylii* in Central Coast streams [24,25]. A large-scale assessment of Bd infections is needed to clarify how infections relate to historical declines in some regions' rivers and persistence in others, identify clusters of increased infection risk across the species’ range, and evaluate how infection incidence and severity changes with the seasonality of the Mediterranean climate and across the diverse ecoregions that *R. boylii* occupies.

Here, we leverage data from over 2000 field and museum samples covering 124 years to synthesize knowledge and evaluate patterns of Bd infections in *R. boylii*. We use a combination of modelling approaches and spatial scan statistics to ask: (i) how are Bd detections in *R. boylii* are distributed over space and time, (ii) whether watersheds with high versus low Bd infection risk clustered historically and today, and (iii) how Bd infections are related to biotic and abiotic factors. Our results highlight priority populations for Bd mitigation, regions that are data-deficient and warrant further sampling and monitoring, and remaining gaps in our knowledge about Bd susceptibility in *R. boylii*. Our study serves as a resource for wildlife managers implementing disease mitigation and species recovery projects, such as re-introductions, and as an example of collaborative research to address conservation challenges in wide-ranging imperilled species.

## 2. Methods

### 2.1. Study species

*Rana boylii* is a stream-dwelling frog (38–81 mm snout–vent length, SVL) [26] whose life cycle timing and breeding migrations between small tributaries and larger main-stem channels occur in synchrony with the winter flood/summer drought flow regime typical of Mediterranean climate rivers. During the transition from high to low flows, adults congregate at lek sites where males reside for weeks to call from underwater, and females visit long enough to mate and attach eggs to rocks in flow-protected locations. Tadpoles reach metamorphosis three–four months later, well before the return of high-flow disturbance the next winter.

**Figure 1.**
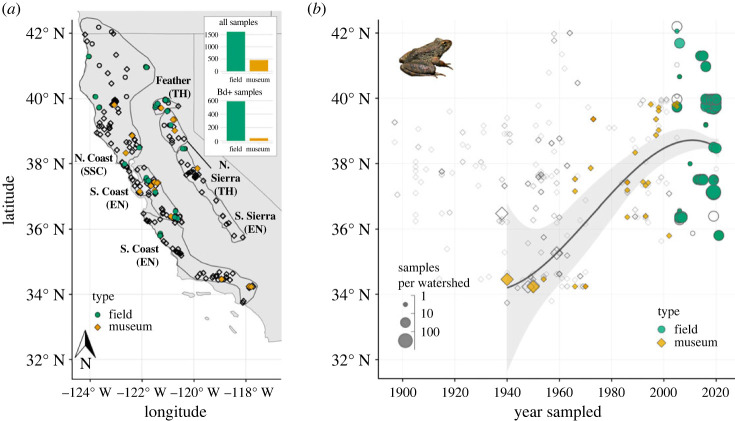
Distribution of *R. boylii* samples assayed for Bd infection. Diamonds show museum samples (collected 1897–2005), circles show field samples (2005–2021). Filled symbols indicate Bd-positive samples. (*a*) Sampling locations across California and Oregon, USA. Symbols overlap in some localities; see inset barplots for sample sizes. *Rana boylii* clades are outlined and labelled, with California Endangered Species Act status abbreviated in parentheses: SSC = Species of Special Concern, TH = Threatened, EN = Endangered. (*b*) Spatio-temporal spread of Bd detections. Symbol size indicates sample size at the HUC-12 (sub-watershed) level. Generalized additive model (GAM) of latitude∼capture year + sample size in Bd-positive samples is shown with black curved line (*R*^2^ = 0.133). Photo of *R. boylii* in Napa County, CA by Marina De León.

These frogs were once common in the rivers and streams flowing through a variety of biomes including moist coniferous forests, oak-savannahs, chaparral and deserts in California and Oregon. Historically, *R. boylii* ranged across at least 12° of latitude, from the Willamette drainage in Oregon to at least the San Gabriel drainage in southern California [26,27], and from sea level to approximately 1500 m [28]. The species tolerates a wide range of conditions, all of which can influence population size and dynamics, including: precipitation that ranges south-to-north from less than 50 to greater than 300 cm yr^−1^ [29]; seasonally variable stream-flow [30]; primary productivity gradients [31]; and thermal regime gradients from rain-dominated coastal watersheds to inland montane (Sierran) snowmelt-driven watersheds [32]. *Rana boylii* comprises six deeply divergent clades that vary in demographic histories, genetic diversity, and California and federal Endangered Species Act protections [33–36] (figure 1*a*).

### 2.2. Data compilation and visualization

To test for the effects of space, time and abiotic and biotic factors on Bd infections in *R. boylii*, we gathered georeferenced Bd data from skin swabs and/or tissues collected from museum specimens or field-sampled frogs from 22 sources (electronic supplementary material, tables S1 and S2). Skin swabs were collected and DNA was extracted from swabs according to a standard protocol [37] with the exception of one museum study that used a DNA extraction method for formalin-fixed specimens [23]. Extracts were analysed with either endpoint or quantitative PCR (qPCR) to detect Bd DNA [38]. Two field-collected samples [39] were analysed using histological examination of skin at the USGS National Wildlife Health Center (Madison, WI). Museum samples were assessed using histology [40], qPCR [12], or a combination of these two techniques [23,24]. Bd assay results and detection method, frog life stage, sex and size were included when available (see electronic supplementary material, methods for additional details on data gathering and quality control). The final dataset included 2145 samples, comprising 461 museum samples of post-metamorphic frogs collected 1897–2005 and 1684 field samples (larval and post-metamorphic frogs) collected 2005–2021. A subset of field-sampled frogs (*n* = 86) were passive integrated transponder (PIT)-tagged and recaptured; a single sample per year was randomly selected for each resampled frog for inclusion in analyses resulting in 2074 total samples and 1613 field samples after filtering (electronic supplementary material, methods). Sampling areas included streams and rivers in 173 sub-watersheds (12-digit USGS hydrologic unit code, HUC-12) across California and Oregon; the diversity of the watersheds is manifest in their spanning the ancestral homelands of at least 41 Indigenous Peoples past, present and future (figure 1*a*, electronic supplementary material, tables S3 and S4).

### 2.3. Analyses of *Batrachochytrium dendrobatidis* detection, prevalence and load over space and time

We tested for a spatio-temporal pattern of Bd detections in museum and field samples using a generalized additive model (GAM; [41]). For the GAM, we grouped samples by HUC-12 and capture year, and filtered the data to only Bd-positive samples. To account for uneven sample sizes across space and time (e.g. no samples collected from southern localities after extirpations in the 1970s), we included total samples (Bd+ and Bd−, *n* = 2074 after filtering recaptures) at each HUC-12 and capture year combination as a fixed effect. We applied a thin plate spline smoothing function to capture year [42]. The GAM was run with the following formula: latitude∼total samples + smoothed capture year.

To identify geographical clusters of Bd infection risk, we used circular spatial scan statistics implemented in SaTScan [43,44]. SaTScan identifies geographical clusters of cases (infections) by calculating relative risk (RR) based on the incidence of infections inside versus outside circular windows. Window size is selected using maximum likelihood, and clusters are designated high or low rate based on RR values (RR > 1 indicating more infections than expected in high-rate clusters, RR < 1 indicating fewer infections than expected in low-rate clusters). We performed separate SaTScan analyses for museum versus field samples. For both we applied SaTScan's Bernoulli model to Bd status (Bd±; [44]). We aggregated Bd detection data at the HUC-12 level to improve cluster detection power in areas with fewer samples [45]), and set maximum window radius to 50 km (the approximate mean HUC-12 size in California). We used minimum high-rate cluster case thresholds of *n* = 3 for museum samples and *n* = 10 for field samples to balance different limitations on detection in formalin-fixed versus field-collected samples with achieving sufficient statistical power in the models (electronic supplementary material, table S5). For cluster selection, we used the following SaTScan settings: Monte Carlo with 999 replications, adjustment for more likely clusters, 10 iterations and alpha = 0.05. We used SaTScan cluster ID and cluster rate (high versus low) in models described below.

### 2.4. Evaluation of *Batrachochytrium dendrobatidis*'s relationships with environmental and host-associated factors

We used generalized linear mixed models (GLMMs) in glmmTMB [46]) to analyze the effects of abiotic and biotic factors on three response variables: Bd detection (Bd+/Bd-) in (i) museum and (ii) field samples, and (iii) infection load (Bd internal transcribed sequence (ITS) copies) in field samples. We first constructed intercept-only models for each of the three response variables with/without random effects of SaTScan cluster ID and cluster rate (0 = not in a cluster, 1 = low-rate cluster, 2 = high-rate cluster). We then selected the best intercept-only model as a base for model building with additional covariates.

We used public databases to compile 85 potentially relevant environmental covariates at the HUC-12 level including hydroclimatic and geographical factors. To identify redundant covariates, we calculated Pearson's *r* for every pair of variables; we selected a single variable from pairs with *r* ≥ 0.7 (electronic supplementary material, tables S6 and S7, and methods).

For the analysis of Bd detection in museum samples, we included only day length, the 24-month standardized precipitation evapotranspiration index (SPEI; a measure of prolonged drought in which negative values indicate more extreme drought and positive values indicate wetter years; hereafter referred to as ‘aridity’), latitude, and decade as covariates; we did not include other covariates as these are based on contemporary measurements and do not reflect the time period of museum collections. We explored season and aridity as interactions with decade, as these factors could not have affected Bd status prior to the earliest Bd detections. We also included interactions with latitude to evaluate how drivers of infection vary in strength regionally.

For the analyses of Bd detection and Bd load in field samples, we first selected the most informative set of non-redundant environmental covariates using a boosted regression tree (BRT) analysis using xgboost in R (v. 3.6.2; [41,47,48]). BRT is well-suited for large complex ecological data as it does not assume normality or linearity; it ignores non-informative predictors and is typically unaffected by outliers; and it can accept numeric, categorical, or binary data [49,50]. Seven covariates surpassed a BRT threshold of approximately 5% relative importance: *T*_max_, latitude, day length, % agriculture on hydric soil, % wetlands remaining, aridity and elevation (electronic supplementary material, figure S1). These covariates were included in the two field sample models.

While spatial and/or temporal variation in environmental covariates are likely, capture year was highly confounded with location in field samples. Therefore, we included interactions between environmental covariates and latitude but not year in the field sample models. We included SVL in the Bd load model based on previously demonstrated relationships [51]. We used log_10_-transformed ITS values for Bd load model analyses.

All covariates were centred and scaled for model building. Alternative models were constructed for each response variable with and without interactions. We used Akaike information criterion (AIC) to select the best model for each response variable.

## 3. Results

### 3.1. Spatial and temporal distribution of *Batrachochytrium dendrobatidis* detections

Sampling was geographically and temporally uneven. Museum specimens made up the majority of South Coast and Southern Sierra samples. Field samples from North Coast and Central Coast clades were concentrated in watersheds that were the focus of research projects or have frog population monitoring programmes stipulated in dam operating licenses (electronic supplementary material, table S3 and figure S2).

We detected a northward spread in Bd detections through time, indicated by a positive curvilinear relationship between year and latitude of Bd detections. Beginning in 2005 when field sampling began, the relationship between year and latitude flattened (GAM, *t* = 4.037, *p* < 0.0001, adjusted *R*^2^ = 0.133, deviance explained = 14.4%; figure 1*b*; electronic supplementary material, figure S3). Bd+ frogs were first detected in 1940 in the Southern California Transverse Range (South Coast clade); in 1966 in the Coast Range (Central Coast), 1972 in the Central California Foothills and Coastal Mountains (Southern Sierra), and 1973 in the Sierra Nevada (Northern Sierra). The earliest North Coast detections were 1989 in the Coast Range (Russian River), 2005 in the Klamath Mountains, and 2016 in the Cascade Mountains.

**Figure 2.**
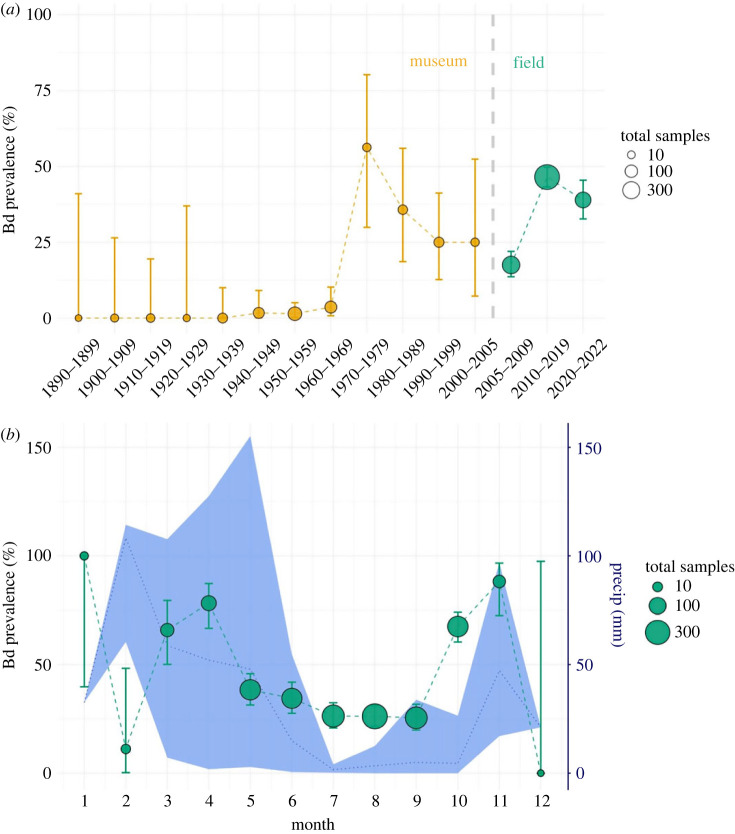
*Batrachochytrium dendrobatidis* infection prevalence (per cent of individuals infected) in foothill yellow-legged frogs (*Rana boylii*) by (*a*) decade across museum and field samples, (*b*) month among field samples only (2005–2021). Museum samples are shown in gold and field samples in green. Error bars are Clopper–Pearson 95% confidence intervals, and dot size is proportional to total sample size. Blue shaded area plot in (*b*) (right y-axis) shows range of monthly precipitation and mean monthly precipitation (dotted line) across all sample sites.

Bd+ individuals occurred throughout the geographical range sampled, with overall prevalence of 8.5% (6.1–11.4% Clopper–Pearson CI) in museum samples and 36.6% (34.2–41.3%) in post-metamorphic field samples. Once detected in 1940, prevalence increased from 2.1% (CI: 0.78–4.6%, *n* = 282) in 1940–1969 to 56.3% (29.9–80.2%, *n* = 16) in 1970–1979. Prevalence dropped from 1980 to 2005 but remained moderately high at 28.6% (CI: 19.2–39.5%, *n* = 84; figure 2*a*).

Bd prevalence in field-sampled *R. boylii* exhibited a seasonal cycle mirroring Mediterranean climate precipitation patterns. Bd prevalence was consistently lower from May to September relative to other months with the exception of October, a dry month with high prevalence (67.4%, CI: 60.3–73.9%; figure 2*b*). The dry season (May–October) comprised 90% of field samples (*n* = 1451/1612). Median Bd loads did not significantly vary across years, but were orders of magnitude higher among frogs caught in the transition months between the wet and dry seasons: April (1.2 × 10^5^ ITS copies), October (2.1 × 10^5^) and November (2.2 × 10^5^) versus all other months (7.7 × 10^2^–7.4 × 10^3^; electronic supplementary material, figure S4).

### 3.2. Spatial clustering of *Batrachochytrium dendrobatidis* infection risk through time

**Figure 3.**
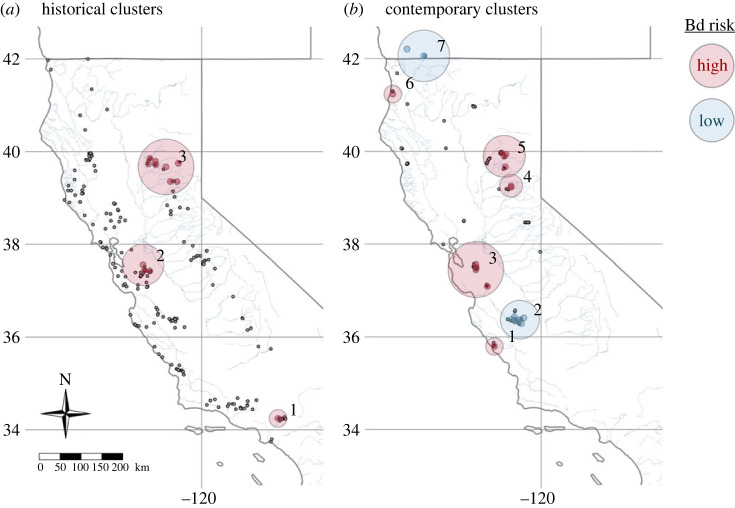
SaTScan clusters of significant relative risk of Bd infection in *Rana boylii*. Samples were aggregated at sub-watershed (HUC-12) level. Historical clusters (*a*) are based on museum samples (collected 1897–2005); contemporary clusters (*b*) are based on field samples (2005–2021). Clusters are shown as large circles, with coloured dots at the centre of sub-watersheds included within a cluster, and small grey dots depicting underlying distribution of aggregated samples. High Bd risk (high-rate) clusters are shown in red (historical clusters 1–3, contemporary clusters 1, 3–6); low Bd risk (low-rate) clusters are shown in blue (contemporary clusters 2, 7). High-rate clusters are defined as areas with significantly higher relative risk than expected (RR > 1), while low-rate clusters have significantly lower relative risk than expected (RR < 1).

Using SaTScan applied to museum samples, we detected three historical high-rate geographical clusters (*n* = 11–17 individuals) spanning four clades: San Gabriel River (sample range 1961–1970, Bd+ samples detected in 1970; South Coast clade), San Francisco Bay Area (1966–1994, Bd+ 1986–1994; Central Coast), and Yuba and Feather Rivers (1952–1998, Bd+ 1973–2005; Northern Sierra and Feather; figure 3*a*, electronic supplementary material, figure S5*a*, electronic supplementary material, table S5).

In our SaTScan analysis of field samples, we detected five contemporary high-rate clusters (*n* = 17–471): San Carpoforo Creek (2021; South Coast), Alameda and Coyote Creeks (2013–2020, Bd+ in 2013–2020; Central Coast), Bear River (2016; Northern Sierra), Yuba and Feather watersheds (2005–2020, Bd+ in 2016–2020; Northern Sierra and Feather), and Redwood Creek (2013–2015, Bd+ in 2014–2015; North Coast); and two low-rate clusters: San Benito watershed (2006–2019, Bd+ in 2006; Central Coast) and Applegate River (2005, North Coast; figure 3*b*, electronic supplementary material, figure S5*b*, electronic supplementary material, table S5).

### 3.3. *Batrachochytrium dendrobatidis* infections across sex, life stage and clade

Bd prevalence was higher in field-sampled males (45.9%, CI: 41.0–50.8%) than in females (35.6%, 30.9–40.5%), but median Bd loads were similar between sexes (2.6 × 10^3^ ITS copies in males versus 2.3 × 10^3^ in females; electronic supplementary material, figure S6). Bd prevalence among tadpoles was 3.9% (1.0–9.7%). While juveniles (40.0%, CI: 35.8–44.3%) exhibited similar prevalence as adults (43.6%, 39.6–47.7%), median Bd loads were higher in juveniles (9.6 × 10^4^ ITS copies versus 4.5 × 10^3^ in adults; electronic supplementary material, figure S6). Bd+ and Bd- frogs did not differ in SVL or weight (electronic supplementary material, figure S7).

Prevalence also varied by clade. Among museum samples, Bd prevalence did not exceed 20% except in Northern Sierra and Feather clades across all decades. Bd prevalence among field-sampled post-metamorphic frogs was moderately high in the South Coast clade (approx. 65%) and northern and central clades (Central Coast, North Coast, Northern Sierra and Feather; 35–56%), but relatively low in the Southern Sierra (14%; electronic supplementary material, figure S8*a* and table S3). Bd loads in field samples were higher in the Central Coast (1.5 × 10^5^ median ITS copies) and Northern Sierra (1.1 × 10^5^) relative to remaining clades (9.0 × 10^2^–4.2 × 10^3^; electronic supplementary material, figure S8*b* and table S3).

### 3.4. Abiotic correlates of *Batrachochytrium dendrobatidis* infections

The best-fit GLMM for museum sample Bd detections included cluster rate as a random intercept; decade as a continuous fixed effect; and one-way interactions between decade and three environmental predictors: day length, aridity and latitude (electronic supplementary material, table S8). Decade was the only significant predictor of Bd detection probability, with a stronger positive relationship between Bd detection probability and time in high-rate clusters relative to samples outside of clusters (decade conditional estimate = 1.54, *p* < 0.0001; electronic supplementary material, figure S9).

**Figure 4.**
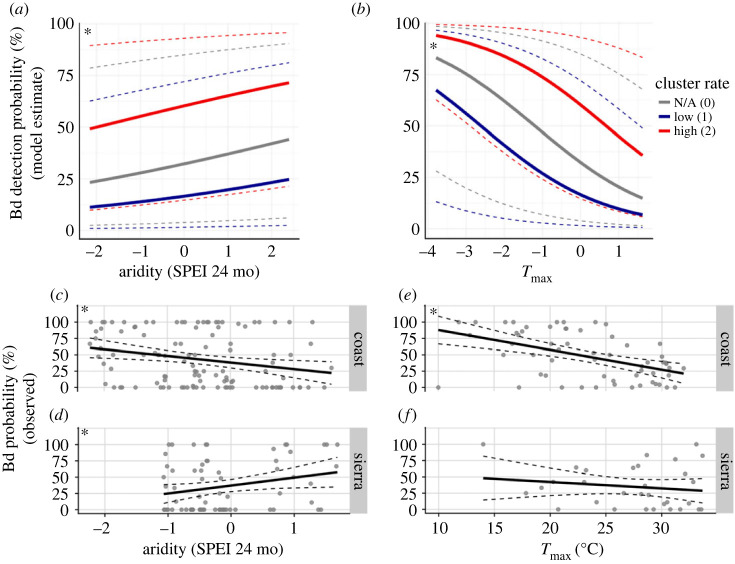
Influence of aridity and air temperature on Bd prevalence in *R. boylii*. Aridity index (SPEI) includes 24 months preceding frog capture; maximum air temperature (*T*_max_) is the maximum over 30 days preceding capture. Asterisks indicate *p* < 0.05. (*a–b*) Marginal effects of aridity (*a*) and *T*_max_ (*b*) on Bd detection probability predicted by GLMM. Red lines and dashed CIs indicate effects in high-rate clusters (cluster rate = 2; figure 3*b*); blue lines and CIs indicate effects in low-rate clusters (rate = 1; figure 3*b*); grey lines and CIs indicate effects in watersheds outside clusters (rate = 0). (*c–f*) Linear regressions of aridity (*c–d*) or *T*_max_ (*e–f*) with observed Bd prevalence (% individuals infected) between rain-driven coastal systems versus inland snowmelt-driven Sierran systems.

The best GLMM for field sample Bd detections included cluster ID and cluster rate as random intercepts; all variables selected by BRT analysis (elevation, day length, latitude, *T*_max_, aridity, % agriculture on hydric soils and % wetlands remaining) as fixed effects; and interactions between latitude and two climatic predictors, *T*_max_ and aridity (electronic supplementary material, table S9). Bd detection probability increased with shorter day length (conditional estimate = −0.16, *p* < 0.05), lower *T*_max_ (i.e, cooler temperatures; conditional estimate = −0.62, *p* < 0.0001) and lower aridity (higher values of SPEI index); conditional estimate = 0.21, *p* < 0.01). The effects of day length, *T*_max_ and aridity on Bd detection probability were marginally stronger in high-rate clusters (figure 4*a,b*, electronic supplementary material, figure S10). Relationships between Bd detection probability and *T*_max_ varied across latitude (latitude–*T*_max_ interaction: conditional estimate = 0.36, *p* < 0.0001). The aridity index showed opposite relationships with observed Bd prevalence in Coastal and Sierran clades: in Coastal clades the relationship was negative (i.e. higher Bd prevalence during prolonged droughts; simple linear regression (SLR), *t* = −10.05, *p* < 0.01, *R*^2 =^ 0.06), while in Sierran clades it was positive (higher Bd prevalence outside of droughts; SLR, *t* = 12.2, *p* < 0.05, *R*^2 =^ 0.06; figure 4*c,d*). Observed Bd prevalence showed a significant negative relationship with *T*_max_ in Coastal clades only (SLR, *t* = −4.15, *p* < 0.001, *R*^2^ = 0.23; figure 4*e,f*).

**Figure 5.**
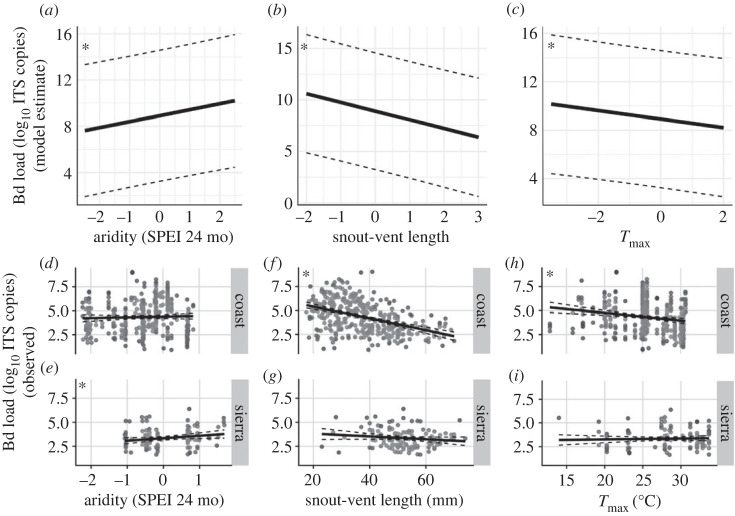
Influence of aridity, body length and air temperature on Bd infection intensity in *R. boylii*. Aridity index (SPEI) includes 24 months preceding frog capture; frog length (SVL) was reported as snout–vent length; maximum air temperature (*T*_max_) was the maximum over 30 days preceding capture. (*a–c*) Marginal effects of aridity (*a*), SVL (*b*), and *T*_max_ (*c*) on Bd loads predicted by GLMM. Grey dashed lines show 95% CIs. Bd loads are reported as log-transformed ITS copy number. Linear regressions of aridity (*d*,*e*), SVL (*f,g*), and *T*_max_ (*h*,*i*) with observed Bd load between rain-driven coastal systems versus inland snowmelt-driven Sierran systems.

The best model for field sample Bd load included cluster ID as a random intercept; *T*_max_, day length, aridity, % agriculture on hydric soils, % wetlands remaining, latitude, elevation and SVL as fixed effects; and no interactions between latitude and other predictors (electronic supplementary material, table S10). Higher Bd loads were associated with lower aridity (conditional estimate = 0.51, *p* < 0.01), smaller SVL (conditional estimate = −0.85, *p* < 0.0001), and lower *T*_max_ (conditional estimate = −0.35, *p* < 0.05). Observed Bd load only showed this inverse relationship with aridity in Sierran clades as evidenced by a positive relationship with the SPEI index (SLR, *t* = 2.2, *p* < 0.05, *R*^2 =^ 0.04), and showed negative relationships with SVL and *T*_max_ in Coastal clades (SVL, *t* = −9.72, *p* < 0.0001, *R*^2^ = 0.20; *T*_max_, *t* = −3.84, *p* < 0.001, *R*^2^ = 0.04; figure 5).

## 4. Discussion

### 4.1. Spatio-temporal patterns in *Batrachochytrium dendrobatidis* infections

We analysed spatio-temporal patterns in Bd detections in *R. boylii* using a compiled dataset comprising over 2000 samples spanning 124 years. Our analyses revealed several notable patterns. First, the 39 Bd+ museum specimens coupled with the earliest Bd detections from the field show a south-to-north progression of detections until 2005 when field swabbing began. Second, Bd prevalence increased through the decades, but the trajectory and timing of peak prevalence varied among climate zones. Among extant populations, those from the most xeric region, California's Central Coast (Central Coast and South Coast clades) had the highest Bd prevalence and infection loads; this region also had some of the earliest Bd detections historically. Third, observed Bd infections varied by frog sex and life stage: Bd prevalence was higher in adult males than females, while loads were highest in juveniles in autumn (primarily from the Central Coast clade). Lastly, we found a strong influence of Mediterranean climate seasonality, whereby both prevalence and loads were lowest during warm and dry summer months. Below we discuss potential implications of these findings, limitations of our analyses and suggestions for future work.

### 4.2. *Batrachochytrium dendrobatidis* dispersal across the US west coast

The earliest Bd detections in *R. boylii* across California and Oregon exhibited a pattern of northward spread corroborated by three historical high-rate clusters: a South Coast cluster in the 1960s–1970s (San Gabriel watershed), a Central Coast cluster in the 1960s–1980s (San Francisco Bay Area watersheds), and a Northern Sierra/Feather River cluster from the 1970s to 2000s (Yuba and Feather watersheds, Sacramento River drainage). Bd prevalence also increased across the decades, rising from less than 5% between 1890 and 1969 to greater than 50% by the 1970s, and staying moderately high (approx. 30%) until the 2000s. Moreover, decade was the only significant predictor of Bd prevalence in museum samples. Taken together, our results suggest that Bd infections in *R. boylii* coincided with historical declines in the South Coast of California before spreading northward across California and Oregon during the middle of the twentieth century. These findings are consistent with previous multi-species museum studies reporting that Bd spread in a wave-like pattern across Central and South America [52–54] and northward in southern California [24,55], and that Bd prevalence increased in California in the 1960s–1970s [12,56,57].

Though our results may suggest directional spread of Bd in *R. boylii*, the ‘Bd wave hypothesis’ may not fully explain the history of chytridiomycosis in our sampling area and neighbouring regions. Previous retrospective studies detected Bd in museum specimens collected decades before reports of chytridiomycosis-associated declines from Baja California, Mexico (1894; [58]), southern California (1915; [23]) and Costa Rica (1964; [57]). Additional evidence suggests that Bd may have been introduced multiple times in California [59] and may have spread from human population centres including the San Francisco Bay Area [40] and the Los Angeles Basin [23]. Time-dated phylogenetic analyses also suggest that Bd may have been present in California for approximately 500–1,400 years before present; however, rapid evolution at high elevation sites may influence these estimates [60].

### 4.3. *Batrachochytrium dendrobatidis* prevalence and load depends on sex and life stage

Behaviour and phenology may explain infection differences among sexes and life stages. Higher prevalence in males may be due to congregating at lek (group breeding) sites and territorial wrestling [61,62] that could increase Bd transmission, as well as reproductive endocrines and stress hormones that can mediate immune function variation among sexes [63–65]. Substantial time spent calling underwater [66] could also increase Bd growth on males, as the fungus is aquatic and psychrophilic (cold-preferring). Water temperatures when *R. boylii* breed are typically cool, averaging 10–12°C [67,68]. Whether males experience increased negative effects of infections is unknown, but in four other California and Oregon ranids, males exhibit greater Bd-associated mortality than females [69]. The observation that juvenile *R. boylii* had the highest pathogen loads may be attributed to the fact that life stage and season are inextricably coupled. Young of the year are abundant in cooler late summer and autumn months when environmental temperatures are similar to the thermal growth optimum for Bd [31,32,70–72].

### 4.4. A *Batrachochytrium dendrobatidis* hotspot in California's Central Coast

Two Central Coast watersheds, Alameda and Coyote Creeks, grouped into a high-rate cluster and exhibited the highest Bd loads. Bd sampling at these sites was triggered by observations of sick and dying frogs in autumn; thus these are not random samples. Nonetheless, factors specific to these watersheds may contribute to greater chytridiomycosis mortality, such as intermittent surface flow during dry seasons that crowds frogs at remnant pools and promotes Bd transmission; frequent human visitation facilitating transmission of multiple Bd strains; and the presence of non-native reservoir/amplification hosts (bullfrogs and crayfish; [24,25,73]). Moreover, the Central Coast clade has especially low genetic diversity [35], which may increase chytridiomycosis susceptibility [74,75].

### 4.5. Do season, temperature and hydrologic conditions drive *Batrachochytrium dendrobatidis* infections?

Shorter day length and lower monthly maximum temperatures were associated with higher Bd infection probability and loads. Our models also indicated Bd infections were associated with successive wet years, but the influence of temperature and aridity varied significantly among clades and latitudes. Moreover, *T*_max_ and aridity serve as reasonable proxies for water temperature and stream-flow, but have limitations. Air and water temperatures in rivers are highly correlated [76], but can be decoupled by factors including stream-bed geomorphology, canopy cover and hypolimnetic releases from upstream impoundments [77–80]. Stream-flows should decrease during droughts, but can be maintained by dam releases [20], intensified by water abstraction [81] and vary with hillslope water storage capacity [82]. Given the significant site-to-site variation in these factors affecting water temperature and stream-flow that could mask the associations of Bd with *T*_max_ and aridity, we interpret the overall relationships as being valid but requiring consideration of local context.

Increasing Bd infections and loads with decreasing temperatures and greater stream-flow is consistent with previous reports of seasonal and latitudinal influences on Bd infection in North America (e.g. [83]) and Bd's low thermal preferences [84]. Yet, the effects of temperature and aridity were region-dependent. Snowmelt-driven Sierran watersheds exhibited higher Bd prevalence in 2017–2018 when precipitation was higher, whereas prevalence was higher in rain-fed coastal watersheds during prolonged drought. Coastal samples were collected during more extreme drought conditions (SPEI < −2) compared with Sierran watersheds (min SPEI = −1), when frogs concentrate in shrinking pools during the cool dry autumn, probably increasing Bd transmission [24,25]. Extreme drought is expected to intensify with climate change across *R. boylii*'s range [85].

### 4.6. Persisting with a deadly pathogen

The range-wide ubiquity of Bd we document here and episodes of chytridiomycosis related die-offs in Central Coast populations [25] raise questions about whether the persistence of robust *R. boylii* populations on the North Coast of California and almost complete extirpation from xeric regions of the South Sierra and South Coast are attributed to differences in Bd susceptibility and/or environmental drivers. Mechanisms associated with variation in Bd susceptibility on the individual level include host genetics; innate, acquired or trans-generational ability to clear infections; anti-Bd function of skin secretions and/or microbiome; and microclimate [74,75,86–88]. Antimicrobial skin peptides (AMPs) have been characterized in *R. boylii* [89,90], but the relationships between AMPs and Bd infection remain to be explored in this species. Cases among the set of recaptured frogs in which infection had cleared (*n* = 18) or load was substantially reduced (*n* = 9) upon recapture 1–3 years later could be due to any of the mechanisms listed above.

Behaviour and environmental conditions probably also play roles in the ability to clear Bd infections. *Rana boylii* is known to bask on river banks and emergent rocks in the stream channel [26,91]. Short-term elevated temperature treatments that mimic basking can inhibit Bd growth [92,93]. Warm and precipitation-free summers with long days provide ample basking opportunities across *R. boylii*'s range, potentially explaining the low summer Bd prevalence that we observed. In autumn, less sunlight reaches canyon-bound streams, resulting in more limited basking opportunities [94,95], which may contribute to increased Bd prevalence in October. Basking opportunities are also dependent on microclimatic conditions, which are mediated by riparian vegetative cover and by flow regulation. Flood suppression by upstream dams allows encroachment of trees into stream channels, whereas wildfire can open the canopy. The Feather River watershed is characterized by a unique combination of dammed and free-flowing streams with frogs present in an area that has experienced intense wildfires [96]. *Rana boylii* is designated as threatened under the US Endangered Species Act [36] in the Feather watershed, and frogs occur at low population densities relative to other regions [97]. Ongoing population monitoring in this region will provide important insights into the relationships between Bd, flow regulation and canopy cover.

On the population level, persistence of *R. boylii* may be attributed to demographic resilience. Population growth rates of *R. boylii* positively correlate with summer stream temperature and are greater following wet years with high total annual volume of stream-flow [97]. The longevity (greater than 10 years) and fecundity of some individuals [30,98], should allow populations to rebound from disease-related losses when instream conditions are favourable for recruitment. At the extreme, one Bd+ female swabbed in 2016 in the Pit River of northern California was recaptured in 2021 (K Breedveld 2021, personal observation).

### 4.7. Limitations and future work

We note some potential limitations of our study. Bd infections increased over time, but lower Bd prevalence in older museum samples may in part be attributed to lower detection probability. DNA-damaging formalin fixation compounded with degradation over time can limit DNA amplification, reducing qPCR detection probability relative to more recently preserved specimens and field-collected swabs [99]. Thus, we cannot conclude definitively that prevalence was lower before 2005, or that the oldest Bd− museum samples truly indicate Bd's absence at a given location. Nonetheless, our samples were collected in similar time periods and regions as multi-species retrospective studies in the region that did detect Bd, and our sample sizes from earlier decades in areas where Bd was not detected in *R. boylii* are relatively robust. Thus, our detection data are likely to provide a reasonable estimate of Bd incidence across *R. boylii* populations over the last century. Another complication in estimating true prevalence is that Bd+ animals may have higher capture probabilities [69,100]; however this bias should be consistent between museum and field samples. In addition, our samples were not a random sample over the annual cycle of high and low flows in rivers that dictate frog activity: the majority of field samples in our dataset were collected during the dry season (May–October), which is the most biologically active period for *R. boylii*, when rivers can safely be waded and frogs can be encountered and caught.

We also note that SaTScan may not be optimal for river-dwelling organisms, as the linear distance-based method may cluster populations not connected by waterways. Sample sizes were uneven across regions and years, potentially causing some level of ascertainment bias; indeed, the contemporary clusters we detected are areas with fairly high *R. boylii* abundance and robust monitoring. However, a well-sampled and frog-abundant watershed (SF Eel River) was not part of a cluster due to a non-significant calculated relative risk. Finally, Bd infections do not necessarily indicate susceptibility to chytridiomycosis. For example, high pathogen loads can result from high vulnerability to Bd or high tolerance, i.e*.* the ability to maintain infection while remaining healthy. Assessing susceptibility requires coupling Bd assays with pathology, mortality/survivorship, or other disease indicators.

Our results suggest several avenues for future work to address Bd risk in *R. boylii*. Research that teases apart relationships between Bd and summer conditions (i.e. warm temperatures associated with reduced infections versus droughts associated with increased frog density and Bd transmission) will be especially informative for conservation. For example, stream restoration projects that aim to connect aquatic habitats could be designed to facilitate reduced disease transmission by considering frog density and thermal conditions, and reintroductions of zoo-reared frogs could be performed at a time of year that minimizes Bd infection risk. As cool temperatures predicted higher Bd infection probability, increased monitoring effort in autumn and spring may improve detection of chytridiomycosis outbreaks. Additional research is also needed to relate Bd-positivity to capture probability in this species, and to relate Bd loads to disease susceptibility among populations, life stages and sexes. Given the dual needs for data on susceptibility and minimizing impacts in remnant populations, we suggest expanding mark–recapture studies into clades designated as ‘Endangered’ (South Coast and South Sierra) and combining these with non-lethal tests of susceptibility such as immunocompetence assays [101], mucosal immunity assays [102], characterization of the microbiome [103–105] and its relationships with temperature across environmental contexts [106,107] and immunogenetics studies [74,75,108].

In addition, Bd genotype data in streams and rivers across California and Oregon are lacking. The global panzootic lineage (Bd-GPL) has been the primary genotype detected in California [60,109–113]. However, Bd-Brazil was detected on three amphibians in Alameda and Santa Clara counties (C Briggs 2019, personal communication; Nayar 2019, unpublished data), where we observed the highest Bd prevalence and loads in *R. boylii*. Virulence can significantly vary among Bd genotypes [114], and plastic gene expression in Bd-GPL can alter virulence [115]. Indeed, Bd-GPL strains collected in northern California exhibit wide variation in virulence [13]. Expanded knowledge of Bd genotype distribution may shed light on the regional differences we observed, and direct future evaluation of Bd's threat to *R. boylii*.

Addressing conservation challenges across large geographical scales requires extensive, multidisciplinary collaboration, especially for aquatic species in arid regions where there are competing demands for water and river flows are often over-allocated [116]. Environmental flow frameworks designed to promote river health by allocating water for ecological purposes [117] would be enhanced by incorporating information on disease. In California, dam operators that generate hydroelectric power, irrigation districts that extract water for agriculture, and municipal utilities that impound rivers to supply drinking water to cities all play important roles in the conservation of riverine biota. There is a need for central reporting and collation of data collected by biologists across sectors including resource agencies, utility companies operating in dammed rivers, and academic scientists and community researchers working in undammed rivers to facilitate rapid knowledge sharing and coordination of responses to disease outbreaks. We present our compilation and analysis of diffuse data as a model for collaborative research on wide-ranging imperilled species.

## Data Availability

Data and relevant code for this research work are stored in GitHub: https://github.com/ryanpeek/rabo_bd_paper/releases/tag/v1.0 and have been archived within the Zenodo repository: https://doi.org/10.5281/zenodo.10436639 [118]. Supplementary material is available online [119].

## References

[1] Bernard RF et al. 2020 Identifying research needs to inform white-nose syndrome management decisions. Conserv. Sci. Pract. **2**, e220. (10.1111/csp2.220)

[2] Schloegel LM, Daszak P, Cunningham AA, Speare R, Hill B. 2010 Two amphibian diseases, chytridiomycosis and ranaviral disease, are now globally notifiable to the World Organization for Animal Health (OIE): An assessment. Dis. Aquat. Organ. **92**, 101-108. (10.3354/dao02140) 21268971

[3] Sleeman JM. 2013 Has the time come for big science in wildlife health? EcoHealth **10**, 335-338. (10.1007/s10393-013-0880-0) 24136386

[4] Olson DH, Aanensen DM, Ronnenberg KL, Powell CI, Walker SF, Bielby J, Garner TWJ, Weaver G, Fisher MC. 2013 Mapping the global emergence of *Batrachochytrium dendrobatidis*, the amphibian chytrid fungus. PLoS ONE **8**, e56802. (10.1371/journal.pone.0056802) 23463502 PMC3584086

[5] Scheele BC et al. 2019 Amphibian fungal panzootic causes catastrophic and ongoing loss of biodiversity. Science **363**, 1459-1463. (10.1126/science.aav0379) 30923224

[6] Lambert MR et al. 2020 Comment on ‘Amphibian fungal panzootic causes catastrophic and ongoing loss of biodiversity’. Science **367**, eaay1838. (10.1126/science.aay1838) 32193293

[7] Corn PS. 2003 Endangered toads in the Rockies. In Symp. on Ecological and Earth Sciences in Mountain Areas, Banff, Canada, 6–10 September 2002, pp. 43-51. Missoula, MT: Aldo Leopold Wilderness Research Institute.

[8] Muths E, Stephen Corn P, Pessier AP, Earl Green D. 2003 Evidence for disease-related amphibian decline in Colorado. Biol. Conserv. **110**, 357-365. (10.1016/S0006-3207(02)00239-2)

[9] Bradley GA, Rosen PC, Sredl MJ, Jones TR, Longcore JE. 2002 Chytridiomycosis in native Arizona frogs. J. Wildl. Dis. **38**, 206-212. (10.7589/0090-3558-38.1.206) 11838218

[10] *Chiricahua Leopard Frog Recovery Plan* (No. E7-10674; pp. 1–149). 2007 USFWS. See https://www.fws.gov/species-publication-action/chiricahua-leopard-frog-recovery-plan.

[11] Jaeger JR, Waddle AW, Rivera R, Harrison DT, Ellison S, Forrest MJ, Vredenburg VT, Van Breukelen F. 2017 *Batrachochytrium dendrobatidis* and the decline and survival of the relict leopard frog. EcoHealth **14**, 285-295. (10.1007/s10393-017-1240-2) 28439781

[12] De León ME, Vredenburg VT, Piovia-Scott J. 2017 Recent emergence of a chytrid fungal pathogen in California cascades frogs (*Rana cascadae*). EcoHealth **14**, 155-161. (10.1007/s10393-016-1201-1) 27957606

[13] Piovia-Scott J et al. 2015 Correlates of virulence in a frog-killing fungal pathogen: evidence from a California amphibian decline. ISME J. **9**, 1570-1578. (10.1038/ismej.2014.241) 25514536 PMC4478697

[14] Vredenburg VT, Knapp RA, Tunstall TS, Briggs CJ. 2010 Dynamics of an emerging disease drive large-scale amphibian population extinctions. Proc. Natl Acad. Sci. USA **107**, 9689-9694. (10.1073/pnas.0914111107) 20457913 PMC2906868

[15] Briggs CJ, Knapp RA, Vredenburg VT. 2010 Enzootic and epizootic dynamics of the chytrid fungal pathogen of amphibians. Proc. Natl Acad. Sci. USA **107**, 9695-9700. (10.1073/pnas.0912886107) 20457916 PMC2906864

[16] Savage AE, Becker CG, Zamudio KR. 2015 Linking genetic and environmental factors in amphibian disease risk. Evol. Appl. **8**, 560-572. (10.1111/eva.12264) 26136822 PMC4479512

[17] Waddle AW, Levy JE, Rivera R, Van Breukelen F, Nash M, Jaeger JR. 2019 Population-level resistance to chytridiomycosis is life-stage dependent in an imperiled anuran. EcoHealth **16**, 701-711. (10.1007/s10393-019-01446-y) 31654279

[18] Lind AJ. 2005 Reintroduction of a declining amphibian: determining an ecologically feasible approach for the foothill yellow-legged frog (*Rana boylii*) through analysis of decline factors, genetic structure, and habitat associations. PhD thesis, University of California, Davis.

[19] Olson DH, Davis RJ. 2009 *Conservation assessment for the foothill yellow-legged frog (Rana boylii) in Oregon*. General Technical Report PSW-GTR-248. United States Department of Agriculture.

[20] Kupferberg S, Palen W, Lind A, Bobzien S, Catenazzi A, Drennan J, Power M. 2012 Effects of flow regimes altered by dams on survival, population declines, and range-wide losses of California river-breeding frogs: flow-regime effects on frogs. Conserv. Biol. **26**, 513-524. (10.1111/j.1523-1739.2012.01837.x) 22594596

[21] Sasso T, Mccallum H, Grogan L. 2021 Occurrence of *Batrachochytrium dendrobatidis* within and between species: a review of influential variables as identified from field studies. Biol. Conserv. **262**, 109300. (10.1016/j.biocon.2021.109300)

[22] Patterson L. 2019 A status review of the foothill yellow-legged frog (Rana boylii) in California. California Department of Fish and Wildlife.

[23] Adams AJ, Pessier AP, Briggs CJ. 2017 Rapid extirpation of a North American frog coincides with an increase in fungal pathogen prevalence: historical analysis and implications for reintroduction. Ecol. Evol. **7**, 10 216-10 232. (10.1002/ece3.3468) PMC5723621 29238549

[24] Adams AJ, Kupferberg SJ, Wilber MQ, Pessier AP, Grefsrud M, Bobzien S, Vredenburg VT, Briggs CJ. 2017 Extreme drought, host density, sex, and bullfrogs influence fungal pathogen infection in a declining lotic amphibian. Ecosphere **8**, e01740. (10.1002/ecs2.1740)

[25] Kupferberg SJ, Moidu H, Adams AJ, Catenazzi A, Grefsrud M, Bobzien S, Leidy R, Carlson SM. 2022 Seasonal drought and its effects on frog population dynamics and amphibian disease in intermittent streams. Ecohydrology **15**, e2395. (10.1002/eco.2395)

[26] Stebbins RC, Mcginnis SM. 2012 Field guide to amphibians and reptiles of California, revised edition (Vol. 103). Berkeley, CA: University of California Press.

[27] Nussbaum RA, Brodie ED, Storm RM. 1983 Amphibians and reptiles of the Pacific Northwest. Caldwell, ID: Caxton Press.

[28] Bedwell ME. 2018 Using genetic tools to investigate distribution and connectivity of two Sierra Nevada amphibians, *Rana sierrae* and *Rana* *boylii*. PhD dissertation, Washington State University.

[29] Iacobellis SF, Cayan DR, Abatzoglou JT et al. 2016 Climate. In Ecosystems of California (eds H Mooney, E Zavaleta), pp. 9-25. Berkeley, CA: University of California Press.

[30] Rose JP, Kupferberg SJ, Wheeler CA, Kleeman PM, Halstead BJ. 2021 Estimating the survival of unobservable life stages for a declining frog with a complex life history. Ecosphere **12**, 2. (10.1002/ecs2.3381)

[31] Catenazzi A, Kupferberg SJ. 2013 The importance of thermal conditions to recruitment success in stream-breeding frog populations distributed across a productivity gradient. Biol. Conserv. **168**, 40-48. (10.1016/j.biocon.2013.09.010)

[32] Catenazzi A, Kupferberg SJ. 2017 Variation in thermal niche of a declining river-breeding frog: from counter-gradient responses to population distribution patterns. Freshw. Biol. **62**, 1255-1265. (10.1111/fwb.12942)

[33] CDFW. 2020 *Notice of Findings for Foothill Yellow-Legged Frog (Rana boylii)*. 13. See https://nrm.dfg.ca.gov/FileHandler.ashx?DocumentID=177905&inline.

[34] Mccartney-Melstad E, Gidiş M, Shaffer HB. 2018 Population genomic data reveal extreme geographic subdivision and novel conservation actions for the declining foothill yellow-legged frog. Heredity **121**, 112-125. (10.1038/s41437-018-0097-7) 29941996 PMC6039490

[35] Peek RA, O'rourke SM, Miller MR. 2021 Flow modification associated with reduced genetic health of a river-breeding frog, *Rana boylii*. Ecosphere **12**, e03496. (10.1002/ecs2.3496)

[36] USFWS. 2023 Endangered and Threatened Wildlife and Plants; Foothill Yellow-Legged Frog; Threatened Status with §4(d) Rule for Two Distinct Population Segments and Endangered Status for Two Distinct Population Segments. 88 Fed. Reg. 59698 (August 29, 2023)

[37] Hyatt AD et al. 2007 Diagnostic assays and sampling protocols for the detection of Batrachochytrium dendrobatidis. Dis. Aquat. Organ. **73**, 175-192. (10.3354/dao073175) 17330737

[38] Boyle DG, Boyle DB, Olsen V, Morgan JAT, Hyatt AD. 2004 Rapid quantitative detection of chytridiomycosis (*Batrachochytrium dendrobatidis*) in amphibian samples using real-time Taqman PCR assay. Dis. Aquat. Organ. **60**, 141-148. (10.3354/dao060141) 15460858

[39] Garcia and Associates. 2010 Feasibility study for the reintroduction of the foothill yellow-legged frog (*Rana boylii*) within the Mt. Tamalpais watershed, Marin County, California.

[40] Padgett-Flohr G, Hopkins R. 2009 Batrachochytrium dendrobatidis, a novel pathogen approaching endemism in central California. Dis. Aquat. Organ. **83**, 1-9. (10.3354/dao02003) 19301630

[41] R Core Team. 2019 R: a language and environment for statistical computing. Vienna, Austria: R Foundation for Statistical Computing.

[42] Wood SN. 2003 Thin plate regression splines. J. R. Stat. Soc. B **65**, 95-114. (10.1111/1467-9868.00374)

[43] Carvalho T, Becker CG, Toledo LF. 2017 Historical amphibian declines and extinctions in Brazil linked to chytridiomycosis. Proc. R. Soc. B **284**, 20162254. (10.1098/rspb.2016.2254) PMC5310605 28179514

[44] Kulldorff M. 2019 *Information Management Services, Inc. SaTScan v9.6: software for the spatial and space-time scan statistics*. See https://www.satscan.org.

[45] Li M, Shi X, Li X, Ma W, He J, Liu T. 2019 Sensitivity of disease cluster detection to spatial scales: an analysis with the spatial scan statistic method. Int. J. Geogr. Inform. Sci. **33**, 2125-2152. (10.1080/13658816.2019.1616741)

[46] Brooks ME, Kristensen K, Benthem KJ, Magnusson A, Berg CW, Nielsen A, Skaug HJ, Machler M, Bolker BM. 2017 GlmmTMB balances speed and flexibility among packages for zero-inflated generalized linear mixed modeling. R J. **9**, 378. (10.32614/RJ-2017-066)

[47] Chen T, Guestrin C. 2016 XGBoost: a scalable tree boosting system. In Proc. of the 22nd ACM SIGKDD Int. Conf. on Knowledge Discovery and Data Mining, San Francisco, CA, 13–17 August, pp. 785-794. New York, NY: ACM.

[48] Kuhn M, Wickham H. 2020 Tidymodels: a collection of packages for modeling and machine learning using tidyverse principles. Boston, MA. See https://www.tidymodels.org/.

[49] Dormann CF et al. 2013 Collinearity: A review of methods to deal with it and a simulation study evaluating their performance. Ecography **36**, 27-46. (10.1111/j.1600-0587.2012.07348.x)

[50] Elith J, Leathwick JR, Hastie T. 2008 A working guide to boosted regression trees. J. Anim. Ecol. **77**, 802-813. (10.1111/j.1365-2656.2008.01390.x) 18397250

[51] Gervasi SS et al. 2017 Linking ecology and epidemiology to understand predictors of multi-host responses to an emerging pathogen, the amphibian chytrid fungus. PloS ONE **12**, e0167882. (10.1371/journal.pone.0167882) 28095428 PMC5240985

[52] Cheng TL, Rovito SM, Wake DB, Vredenburg VT. 2011 Coincident mass extirpation of neotropical amphibians with the emergence of the infectious fungal pathogen *Batrachochytrium dendrobatidis*. Proc. Natl Acad. Sci. USA **108**, 9502-9507. (10.1073/pnas.1105538108) 21543713 PMC3111304

[53] Lips KR et al. 2006 Emerging infectious disease and the loss of biodiversity in a Neotropical amphibian community. Proc. Natl Acad. Sci. USA **103**, 3165-3170. (10.1073/pnas.0506889103) 16481617 PMC1413869

[54] Velo-Antón G, Rodríguez D, Savage AE, Parra-Olea G, Lips KR, Zamudio KR. 2012 Amphibian-killing fungus loses genetic diversity as it spreads across the New World. Biol. Conserv. **146**, 213-218. (10.1016/j.biocon.2011.12.003)

[55] Chaukulkar S, Sulaeman H, Zink AG, Vredenburg VT. 2018 Pathogen invasion and non-epizootic dynamics in Pacific newts in California over the last century. PloS ONE **13**, e0197710. (10.1371/journal.pone.0197710) 29965970 PMC6028104

[56] Sette CM, Vredenburg VT, Zink AG. 2015 Reconstructing historical and contemporary disease dynamics: a case study using the California slender salamander. Biol. Conserv. **192**, 20-29. (10.1016/j.biocon.2015.08.039)

[57] De León ME, Zumbado-Ulate H, García-Rodríguez A, Alvarado G, Sulaeman H, Bolaños F, Vredenburg VT. 2019 *Batrachochytrium dendrobatidis* infection in amphibians predates first known epizootic in Costa Rica. PLoS ONE **14**, e0208969. (10.1371/journal.pone.0208969) 31821326 PMC6903748

[58] Basanta MD, Byrne AQ, Rosenblum EB, Piovia-Scott J, Parra-Olea G. 2021 Early presence of Batrachochytrium dendrobatidis in Mexico with a contemporary dominance of the global panzootic lineage. Mol. Ecol. **30**, 424-437. (10.1111/mec.15733) 33205419

[59] Vredenburg VT et al. 2020 Pathogen invasion history elucidates contemporary host pathogen dynamics. PLoS ONE **14**, e0219981. (10.1371/journal.pone.0219981) PMC6752790 31536501

[60] Rothstein AP, Byrne AQ, Knapp RA, Briggs CJ, Voyles J, Richards-Zawacki CL, Rosenblum EB. 2021 Divergent regional evolutionary histories of a devastating global amphibian pathogen. Proc. R. Soc. B **288**, 20210782. (10.1098/rspb.2021.0782) PMC8220259 34157877

[61] Wheeler CA, Welsh HH. 2008 Mating strategy and breeding patterns of the foothill yellow-legged frog (*Rana boylii*). Herpetol. Conserv. Biol. **3**, 128-142.

[62] Wilcox JT, Alvarez JA. 2019 Wrestling for real estate: male-male interactions in breeding foothill yellow-legged frogs (*Rana boylii*). Western Wildlife **6**, 14-17.

[63] Fonner CW, Patel SA, Boord SM, Venesky MD, Woodley SK. 2017 Effects of corticosterone on infection and disease in salamanders exposed to the amphibian fungal pathogen *Batrachochytrium dendrobatidis*. Dis. Aquat. Organ. **123**, 159-171. (10.3354/dao03089) 28262636

[64] Kindermann C, Narayan E, Hero J-M. 2017 Does physiological response to disease incur cost to reproductive ecology in a sexually dichromatic amphibian species? Comp. Biochem. Physiol. A: Mol. Integr. Physiol. **203**, 220-226. (10.1016/j.cbpa.2016.09.019) 27712921

[65] Rollins-Smith LA, Ramsey JP, Pask JD, Reinert LK, Woodhams DC. 2011 Amphibian immune defenses against chytridiomycosis: impacts of changing environments. Integr. Comp. Biol. **51**, 552-562. (10.1093/icb/icr095) 21816807

[66] Silver CS. 2018 Population-level variation in vocalizations of *Rana boylii*, the foothill yellow-legged frog. MS thesis, Chico State University, Chico, CA.

[67] Kupferberg SJ. 1996 Hydrologic and geomorphic factors affecting conservation of a river-breeding frog (*Rana boylii*). Ecol. Appl. **6**, 1332-1344. (10.2307/2269611)

[68] Wheeler CA, Lind AJ, Welsh HH, Cummings AK. 2018 Factors that influence the timing of calling and oviposition of a lotic frog in northwestern California. J. Herpetol. **52**, 289-298. (10.1670/17-103)

[69] Russell RE et al. 2019 Effect of amphibian chytrid fungus (*Batrachochytrium dendrobatidis*) on apparent survival of frogs and toads in the western USA. Biol. Conserv. **236**, 296-304. (10.1016/j.biocon.2019.05.017)

[70] Bondi CA, Yarnell SM, Lind AJ. 2013 Transferability of habitat suitability criteria for a stream breeding frog (*Rana boylii*) in the Sierra Nevada, California. Herpetol. Conserv. Biol. **8**, 88-103.

[71] Piotrowski JS, Annis SL, Longcore JE. 2004 Physiology of *Batrachochytrium dendrobatidis*, a chytrid pathogen of amphibians. Mycologia **96**, 9-15. (10.1080/15572536.2005.11832990) 21148822

[72] Woodhams DC, Alford RA, Briggs CJ, Johnson M, Rollins-Smith LA. 2008 Life-history trade-offs influence disease in changing climates: strategies of an amphibian pathogen. Ecology **89**, 1627-1639. (10.1890/06-1842.1) 18589527

[73] Mcmahon TA, Brannelly LA, Chatfield MW, Johnson PT, Joseph MB, Mckenzie VJ, Rohr JR. 2013 Chytrid fungus *Batrachochytrium dendrobatidis* has nonamphibian hosts and releases chemicals that cause pathology in the absence of infection. Proc. Natl Acad. Sci. USA **110**, 210-215. (10.1073/pnas.1200592110) 23248288 PMC3538220

[74] Belasen AM, Amses KR, Clemons RA, Becker CG, Toledo LF, James TY. 2022 Habitat fragmentation in the Brazilian Atlantic Forest is associated with erosion of frog immunogenetic diversity and increased fungal infections. Immunogenetics **74**, 431-441. (10.1007/s00251-022-01252-x) 35080658 PMC11344651

[75] Savage AE, Zamudio KR. 2011 MHC genotypes associate with resistance to a frog-killing fungus. Proc. Natl Acad. Sci. USA **108**, 16 705-16 710. (10.1073/pnas.1106893108) PMC3189034 21949385

[76] Steel EA, Beechie TJ, Torgersen CE, Fullerton AH. 2017 Envisioning, quantifying, and managing thermal regimes on river networks. BioScience **67**, 506-522. (10.1093/biosci/bix047)

[77] Kędra M, Wiejaczka Ł. 2018 Climatic and dam-induced impacts on river water temperature: assessment and management implications. Sci. Total Environ. **626**, 1474-1483. (10.1016/j.scitotenv.2017.10.044) 29074247

[78] Mihalevich BA, Neilson BT, Buahin CA, Yackulic CB, Schmidt JC. 2020 Water temperature controls for regulated canyon-bound rivers. Water Resour. Res. **56**, e2020WR027566. (10.1029/2020WR027566)

[79] Olden JD, Naiman RJ. 2010 Incorporating thermal regimes into environmental flows assessments: modifying dam operations to restore freshwater ecosystem integrity: Incorporating thermal regimes in environmental flows assessments. Freshw. Biol. **55**, 86-107. (10.1111/j.1365-2427.2009.02179.x)

[80] Woltemade CJ. 2017 Stream temperature spatial variability reflects geomorphology, hydrology, and microclimate: Navarro River Watershed, California. Professional Geogr. **69**, 177-190. (10.1080/00330124.2016.1193032)

[81] Dillis C, Mcintee C, Butsic V, Le L, Grady K, Grantham T. 2020 Water storage and irrigation practices for cannabis drive seasonal patterns of water extraction and use in Northern California. J. Environ. Manage. **272**, 110955. (10.1016/j.jenvman.2020.110955) 32677619

[82] Hahm WJ, Dralle DN, Rempe DM, Bryk AB, Thompson SE, Dawson TE, Dietrich WE. 2019 Low subsurface water storage capacity relative to annual rainfall decouples Mediterranean plant productivity and water use from rainfall variability. Geophys. Res. Lett. **46**, 6544-6553. (10.1029/2019GL083294)

[83] Sonn JM, Utz RM, Richards-Zawacki CL. 2019 Effects of latitudinal, seasonal, and daily temperature variations on chytrid fungal infections in a North American frog. Ecosphere **10**, e02892. (10.1002/ecs2.2892)

[84] Lindauer AL, Maier PA, Voyles J. 2020 Daily fluctuating temperatures decrease growth and reproduction rate of a lethal amphibian fungal pathogen in culture. BMC Ecol. **20**, 18. (10.1186/s12898-020-00286-7) 32245440 PMC7118903

[85] Swain DL. 2021 A shorter, sharper rainy season amplifies California wildfire risk. Geophys. Res. Lett. **48**, e2021GL092843. (10.1029/2021GL092843)

[86] Jiménez RR, Carfagno A, Linhoff L, Gratwicke B, Woodhams DC, Chafran LS, Bletz MC, Bishop B, Muletz-Wolz CR. 2022 Inhibitory bacterial diversity and mucosome function differentiate susceptibility of Appalachian salamanders to chytrid fungal infection. Appl. Environ. Microbiol. **88**, e01818-21. (10.1128/aem.01818-21) 35348389 PMC9040618

[87] Roth O, Beemelmanns A, Barribeau SM, Sadd BM. 2018 Recent advances in vertebrate and invertebrate transgenerational immunity in the light of ecology and evolution. Heredity **121**, 225-238. (10.1038/s41437-018-0101-2) 29915335 PMC6082847

[88] Zumbado-Ulate H, Bolaños F, Gutiérrez-Espeleta G, Puschendorf R. 2014 Extremely low prevalence of *Batrachochytrium dendrobatidis* in frog populations from Neotropical dry forest of Costa Rica supports the existence of a climatic refuge from disease. EcoHealth **11**, 593-602. (10.1007/s10393-014-0967-2) 25212725

[89] Conlon JM, Sonnevend A, Patel M, Davidson C, Nielsen PF, Pal T, Rollins-Smith LA. 2003 Isolation of peptides of the brevinin-1 family with potent candidacidal activity from the skin secretions of the frog *Rana boylii*: antimicrobial peptides from *Rana boylii*. J. Peptide Res. **62**, 207-213. (10.1034/j.1399-3011.2003.00090.x) 14531844

[90] Robertson LS, Fellers GM, Marranca JM, Kleeman PM. 2013 Expression analysis and identification of antimicrobial peptide transcripts from six North American frog species. Dis. Aquat. Organ. **104**, 225-236. (10.3354/dao02601) 23759560

[91] Leidy RA, Gonsolin E, Leidy GA. 2009 Late-summer aggregation of the foothill yellow-legged frog (*Rana boylii*) in central California. Southwestern Naturalist **54**, 367-368. (10.1894/WL-21.1)

[92] Daskin JH, Alford RA, Puschendorf R. 2011 Short-term exposure to warm microhabitats could explain amphibian persistence with *Batrachochytrium dendrobatidis*. PLoS ONE **6**, e26215. (10.1371/journal.pone.0026215) 22028834 PMC3196517

[93] Stevenson LA, Roznik EA, Greenspan SE, Alford RA, Pike DA. 2020 Host thermoregulatory constraints predict growth of an amphibian chytrid pathogen (*Batrachochytrium dendrobatidis*). J. Therm. Biol **87**, 102472. (10.1016/j.jtherbio.2019.102472) 31999604

[94] Bode CA, Limm MP, Power ME, Finlay JC. 2014 Subcanopy solar radiation model: predicting solar radiation across a heavily vegetated landscape using LiDAR and GIS solar radiation models. Remote Sens. Environ. **154**, 387-397. (10.1016/j.rse.2014.01.028)

[95] Savoy P, Bernhardt E, Kirk L, Cohen MJ, Heffernan JB. 2021 A seasonally dynamic model of light at the stream surface. Freshw. Sci. **40**, 286-301. (10.1086/714270)

[96] Brewer MJ, Clements CB. 2019 The 2018 Camp fire: meteorological analysis using *in situ* observations and numerical simulations. Atmosphere **11**, 47. (10.3390/atmos11010047)

[97] Rose JP, Kupferberg SJ, Peek RA, Ashton D, Bettaso JB, Bobzien S, Halstead BJ. 2023 Identifying drivers of population dynamics for a stream breeding amphibian using time series of egg mass counts. Ecosphere **14**, e4645. (10.1002/ecs2.4645)

[98] Marlow K, Wiseman K, Wheeler C, Drennan J, Jackman RE. 2016 Identification of individual foothill yellow-legged frogs (Rana boylii) using chin pattern photographs: a non-invasive and effective method for small population studies. *Herpetol. Rev.* **47**, 193–198.

[99] Adams AJ, Labonte JP, Ball ML, Richards-Hrdlicka KL, Toothman MH, Briggs CJ. 2015 DNA extraction method affects the detection of a fungal pathogen in formalin-fixed specimens using qPCR. PLoS ONE **10**, e0135389. (10.1371/journal.pone.0135389) 26291624 PMC4546330

[100] Joseph MB, Knapp RA. 2018 Disease and climate effects on individuals drive post-reintroduction population dynamics of an endangered amphibian. Ecosphere **9**, e02499. (10.1002/ecs2.2499)

[101] Savage AE, Terrell KA, Gratwicke B, Mattheus NM, Augustine L, Fleischer RC. 2016 Reduced immune function predicts disease susceptibility in frogs infected with a deadly fungal pathogen. Conservation Physiology **4**, cow011. (10.1093/conphys/cow011) 27293759 PMC4834730

[102] Woodhams DC et al. 2014 Interacting Symbionts and Immunity in the Amphibian Skin Mucosome Predict Disease Risk and Probiotic Effectiveness. PLoS ONE **9**, e96375. (10.1371/journal.pone.0096375) 24789229 PMC4005770

[103] Bletz MC et al. 2017 Amphibian skin microbiota exhibits temporal variation in community structure but stability of predicted Bd-inhibitory function. ISME J. **11**, 1521-1534. (10.1038/ismej.2017.41) 28387770 PMC5520157

[104] Jani AJ, Bushell J, Arisdakessian CG, Belcaid M, Boiano DM, Brown C, Knapp RA. 2021 The amphibian microbiome exhibits poor resilience following pathogen-induced disturbance. ISME J. **15**, 1628-1640. (10.1038/s41396-020-00875-w) 33564111 PMC8163836

[105] Piovia-Scott J, Rejmanek D, Woodhams DC, Worth SJ, Kenny H, Mckenzie V, Lawler SP, Foley JE. 2017 Greater species richness of bacterial skin symbionts better suppresses the amphibian fungal pathogen *Batrachochytrium dendrobatidis*. Microb. Ecol. **74**, 217-226. (10.1007/s00248-016-0916-4) 28064360

[106] Longo AV, Zamudio KR. 2017 Temperature variation, bacterial diversity and fungal infection dynamics in the amphibian skin. Mol. Ecol. **26**, 4787-4797. (10.1111/mec.14220) 28664981

[107] Muletz-Wolz CR, Fleischer RC, Lips KR. 2019 Fungal disease and temperature alter skin microbiome structure in an experimental salamander system. Mol. Ecol. **28**, mec.15122. (10.1111/mec.15122) 31066947

[108] Bataille A et al. 2015 Susceptibility of amphibians to chytridiomycosis is associated with MHC class II conformation. Proc. R. Soc. B **282**, 20143127. (10.1098/rspb.2014.3127) PMC4389617 25808889

[109] Byrne AQ et al. 2019 Cryptic diversity of a widespread global pathogen reveals expanded threats to amphibian conservation. Proc. Natl Acad. Sci. USA **116**, 20 382-20 387. (10.1073/pnas.1908289116) 31548391 PMC6789904

[110] James TY et al. 2009 Rapid global expansion of the fungal disease chytridiomycosis into declining and healthy amphibian populations. PLoS Pathog. **5**, e1000458. (10.1371/journal.ppat.1000458) 19478871 PMC2680619

[111] Rosenblum EB et al. 2013 Complex history of the amphibian-killing chytrid fungus revealed with genome resequencing data. Proc. Natl Acad. Sci. USA **110**, 9385-9390. (10.1073/pnas.1300130110) 23650365 PMC3677446

[112] Schloegel LM et al. 2010 The North American bullfrog as a reservoir for the spread of Batrachochytrium dendrobatidis in Brazil: The North American bullfrog as a reservoir for the spread of an amphibian pathogen. Animal Conservation **13**, 53-61. (10.1111/j.1469-1795.2009.00307.x)

[113] Schloegel LISAM et al. 2012 Novel, panzootic and hybrid genotypes of amphibian chytridiomycosis associated with the bullfrog trade. Mol. Ecol. **21**, 5162-5177. (10.1111/j.1365-294X.2012.05710.x) 22857789

[114] Belasen AM, Russell ID, Zamudio KR, Bletz MC. 2022 Endemic lineages of *Batrachochytrium dendrobatidis* are associated with reduced chytridiomycosis-induced mortality in amphibians: evidence from a meta-analysis of experimental infection studies. Front. Vet. Sci. **9**, 15. (10.3389/fvets.2022.756686) PMC8931402 35310410

[115] Torres-Sánchez M, Longo AV. 2022 Linking pathogen–microbiome–host interactions to explain amphibian population dynamics. Mol. Ecol. **31**, 5784-5794. (10.1111/mec.16701) 36130047

[116] Grantham TE, Viers JH. 2014 100 years of California's water rights system: patterns, trends and uncertainty. Environ. Res. Lett. **9**, 084012. (10.1088/1748-9326/9/8/084012)

[117] Stein ED et al. 2021 The California environmental flows framework: meeting the challenges of developing a large-scale environmental flows program. Frontiers in Environmental Science **9**, 769943. (10.3389/fenvs.2021.769943)

[118] Peek R. 2023 Code for: ryanpeek/rabo_bd_paper: Chytrid infections exhibit historical spread and contemporary seasonality in a declining stream-breeding frog (v1.0). Zenodo. (10.5281/zenodo.10436639) PMC10827429 38298390

[119] Belasen AM et al. 2024 Chytrid infections exhibit historical spread and contemporary seasonality in a declining stream-breeding frog. Figshare. (10.6084/m9.figshare.c.7041551)

